# Obesity-Related Asthma: Immune Regulation and Potential Targeted Therapies

**DOI:** 10.1155/2018/1943497

**Published:** 2018-06-28

**Authors:** Yuze Yuan, Nan Ran, Lingxin Xiong, Guoqiang Wang, Xuewa Guan, Ziyan Wang, Yingqiao Guo, Zhiqiang Pang, Keyong Fang, Junying Lu, Chao Zhang, Ruipeng Zheng, Jingtong Zheng, Jie Ma, Fang Wang

**Affiliations:** ^1^Department of Pathogeny Biology, College of Basic Medical Sciences, Jilin University, Changchun 130021, China; ^2^School of Pharmaceutical Sciences, Jilin University, Changchun 130021, China; ^3^Department of Intensive Care Unit, First Hospital of Jilin University, Changchun 130021, China; ^4^Department of Interventional Therapy, First Hospital of Jilin University, Changchun 130021, China

## Abstract

Obesity, one of the most severe public health problems of the 21st century, is a common metabolic syndrome due to excess body fat. The incidence and severity of obesity-related asthma have undergone a dramatic increase. Because obesity-related asthma is poorly controlled using conventional therapies, alternative and complementary therapies are urgently needed. Lipid metabolism may be abnormal in obesity-related asthma, and immune modulation therapies need to be investigated. Herein, we describe the immune regulators of lipid metabolism in obesity as well as the interplay of obesity and asthma. These lay the foundations for targeted therapies in terms of direct and indirect immune regulators of lipid metabolism, which ultimately help provide effective control of obesity-related asthma with a feasible treatment strategy.

## 1. Introduction

Obesity is defined as an excess of body fat, and it is one of the main public health challenges worldwide. It increases the risk for certain diseases and disorders, including type 2 diabetes, hypertension, chronic kidney disease, cardiovascular diseases, certain types of cancer, and depression [1]. According to a previous report, approximately 13% of adults worldwide are obese [2]. In 2011–2014, 17% of people aged 2 to 19 years in the US were obese, and in 2011-2012, 38% were either overweight or obese; these are substantial increases in the past three decades [3]. Obesity in the US accounts for up to one-third of total mortality. A study from the Global Burden of Disease (GBD) revealed that the global obesity epidemic is worsening and is placing heavy public health and economic burdens in most regions of this planet. Thus, effective treatments for controlling obesity is necessary [1, 4].

Asthma is a chronic inflammatory disease characterized by variable symptoms of wheezing, shortness of breath, chest tightness, and/or cough and by variable expiratory airflow limitation. It is triggered by multiple factors such as exercise, allergen or irritant exposure, change in weather, or viral respiratory infections [5]. Previous studies have shown that obesity increases the severity of asthma, which demonstrates the close association between these two conditions [6, 7]. Global epidemiological studies on asthma and obesity have also shown that obesity-related asthma has reached an alarming level [8]. Asthma in obese patients is poorly controlled using standard asthma medications including oral corticosteroids, which is partial because the underlying metabolic mechanism, immune cells, and proteins involved in related signaling pathways may be unresponsive to corticosteroids [9]. Hence, additional treatments are urgently needed for the treatment of obesity-related asthma.

We have not yet reached a consensus concerning the accurate and comprehensive pathogenesis of obesity. However, lipid metabolism, which is a major part of energy homeostasis, is undoubtedly involved in the onset and development of obesity [4].

In this review, we summarize the association among lipid metabolism, obesity, and asthma. We also detail the roles of immune responses in lipid metabolism and the pathogenesis of obesity-related asthma. Ultimately, we propose various potential targeted therapies according to distinct cellular types and proteins involved in the regulation of lipid metabolism in obesity-related asthma.

## 2. Immune Modulators of Lipid Metabolism in Obesity

### 2.1. Sterol Regulatory Element-Binding Proteins and Lipid Metabolism

Sterol regulatory element-binding proteins (SREBPs) are significant transcription factors that regulate lipid biosynthesis [10]. This keeps the balance of cholesterol and fatty acids through the activation of gene-encoding enzymes [11]. Additionally, SREBPs play an important role as an interchange node within global signaling networks in a variety of physiological and pathophysiological processes [12]. SREBPs also improve the gene expression of low-density lipoprotein (LDL) receptors (LDLR), which are involved in sterol regulation [13–15]. SREBPs are divided into different isoforms, including SREBP1a, SREBP1c, and SREBP2. The physiological roles of SREBPs vary. SREBP1a is involved in global lipid synthesis and growth, SREBP1c is involved in fatty acid synthesis and energy storage, and SREBP2 is involved in the regulation of cholesterol synthesis [16]. Moreover, SREBPs are involved in a myriad of cellular processes and pathologies such as reactive oxygen species (ROS) production, endoplasmic reticulum stress, apoptosis, and autophagy [17]. Nevertheless, the underlying molecular mechanisms remain unclear and need further studies. SREBP1a might modulate the innate immune responses of macrophages, whereas SREBP2 is associated with cell phagocytosis and autophagy, indicating the significant role of SREBPs in the onset and development of chronic inflammatory diseases such as obesity [18–20]. These findings also suggest that targeting SREBPs may be clinically feasible and promising in the treatment of obesity.

### 2.2. Dipeptidyl Peptidase-4 and Lipid Metabolism

Dipeptidyl peptidase-4 (DPP-4), also known as T-cell surface marker CD26, is widely expressed in multiple cells, particularly in immune cells [20]. It cleaves various chemokines and peptide hormones involved in the regulation of immune response, and it plays an important role in the pathogenesis of inflammation [21, 22]. Previous studies reported on the function of DPP-4, which serves as a surface protease in T-cell activation [23]. Rufinatscha et al. found that in DPP-4 knockdown cells, the levels of triglyceride and peroxisome proliferator-activated receptor alpha (PPAR*α*) were increased, while SREBP-1c expression was obviously decreased [24]. Similarly, Mulvihill found that inhibition of DPP4 significantly reduced postprandial lipoprotein secretion [25]. For example, one selective DPP-4 inhibitor, vildagliptin, led to a significant reduction in total triglyceride and apolipoprotein B-48 (apoB48) concentrations after a high-fat meal [26]. Recent studies showed that another DPP-4 inhibitor, anagliptin, could significantly decrease the expression level of SREBP2 messenger ribonucleic acid, which significantly decreased the plasma total cholesterol and triglyceride levels in anagliptin-treated mice. Both low-density lipoprotein cholesterol and very low-density lipoprotein cholesterol levels were also decreased significantly [27].

### 2.3. Nuclear Factor- (Erythroid-Derived 2-) Like2 and Lipid Metabolism

In addition to SREBP and DPP-4, nuclear factor- (erythroid-derived 2-) like2 (Nrf2), a basic leucine zipper transcription factor, is widely expressed in human and mouse tissues as a defense against exogenous and endogenous stimulation [28]. Obesity is a low-grade inflammatory disorder, and in a previous investigation, Nrf2 was reported to be involved in antiobesity activity. Nrf2 inducers mitigated the weight gain, insulin resistance, oxidative stress, and chronic inflammation induced by a high-fat diet (HFD) in mice [29]. In the same study, the weight-reducing and insulin-sensitizing effects of Nrf2 inducers were abrogated in Nrf2^−/−^ mice, which indicates the importance of Nrf2 in host antiobesity activity. Consistent with the findings of this study, the Nrf2 inducer glucoraphanin increased energy expenditure, decreased lipid peroxidation, and activated M1-like macrophage accumulation and inflammation signaling in HFD-fed mice. Further experiments uncovered that glucoraphanin mitigates obesity by promoting fat browning, limiting metabolic endotoxemia-related chronic inflammation, and regulating redox stress, which suggests that Nrf2-targeted therapy may be clinically promising in the treatment of obesity [29].

### 2.4. Intestinal Microbiota and Lipid Metabolism

Bacterial bile salt hydrolase (BSH) enzymes in the gut play a significant role in the metabolism of bile acids [30]. Joyce et al. found that weight gain of mice with normal microbiota and the level of serum cholesterol and liver triglycerides are reduced by the expression of BSH, which indicated that using BSH may regulate host lipid metabolism [31]. A study showed that the levels of plasma triglycerides and muscle lipid (triglycerides and phospholipids) were significantly decreased in mice fed a diet with prebiotic compared to those that were fed a control diet. Additionally, they also found that the expression of muscle lipoprotein lipase mRNA increased in mice with prebiotic treatment, which may have resulted in the decrease in levels of plasma and muscle lipid [32]. Many studies have demonstrated that HFD feeding profoundly affects the gut microbial community [33–35]. Marc Schneeberger's research indicated that the number of *Akkermansia muciniphila* was reduced in mice with HFD treatment, which was significantly and positively correlated with fatty acid oxidation and browning. However, it was negatively associated with lipid synthesis, adiposity, and inflammatory markers [36]. The main route of cholesterol excretion is the conversion of cholesterol into bile acid in the liver, followed by excretion of bile acids through the feces. Bile is also considered to be bacteriostatic and to prevent overgrowth of small intestinal bacteria [37].

Control bacterial fermentation of dietary fiber in the colon produced short-chain fatty acids (SCFAs). SCFAs regulated the proliferation and apoptosis of cells, which affected intestinal permeability. Additionally, SCFAs could regulate anti-inflammatory effects on the intestinal epithelium by serving as ligands for a series of G-protein-coupled receptors (GPRs) [38]. Experiments have shown that SCFA levels are higher in feces of obese (ob/ob) mice and obese human subjects, which may be due to the reduction of colonic absorption of SCFA leading to obesity [39].

## 3. Obesity and Asthma

**Figure 1:**
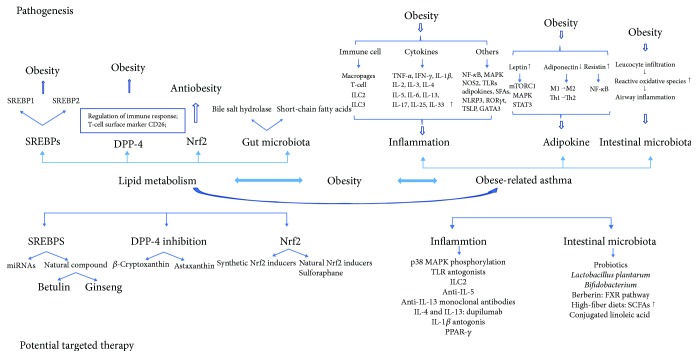
Possible connections among lipid metabolism, obesity, and obesity-related asthma. This figure is divided into two parts: the upper part about pathogenesis and the lower part about potential targeted therapy. The part on pathogenesis demonstrates a few major effects of immune regulators. The other introduces the corresponding possible targeted therapy including SREBPs, miRNAs, and the DPP-4 inhibitor.

In recent years, the incidences of obesity and asthma have been rising with a parallel relationship. The presence of obesity increases the risk for several diseases including asthma [40–42]. Asthma is more common in obese than in nonobese people [43]. Studies have demonstrated that people with asthma have higher BMIs than those without asthma. [44] Moreover, some obese patients with asthma have significant respiratory symptoms and little eosinophilic airway inflammation. Similarly, concordant and growing evidence also supports the relationship between being overweight (defined by body mass index (BMI)) and having asthma [40–42]. Obesity has emerged as a serious risk factor for bronchial asthma, which indicates that obesity could cause or even worsen asthma. Additionally, asthma is more difficult to control in obese patients [45]. Therefore, this study focused on obesity-related asthma. Potential factors that affect the pathogenesis of obesity-related asthma are also summarized in the review (Figure 1).

### 3.1. Low-Grade Inflammation and Asthma

Obesity is regarded as an inflammatory disease [46]. Unlike typical inflammation, it is chronic low-grade systemic sterile inflammation that is characterized by only moderate upregulation of circulating proinflammatory factors and the absence of clinical symptoms of inflammation [47]. The inflammatory response may affect pulmonary function and thus worsen the asthma. In particular, macrophages play an important role in the occurrence and development of obesity-associated asthma. The HFD mice in obesity modelling increase the number of macrophages in the lungs and alveoli. Besides total cell count in bronchoalveolar lavage fluid (BALF), neutrophils and a few eosinophils also had increased counts [48]. In addition, the concentration of Th1 cytokines and IFN-*γ* also increased significantly in BALF [49]. In healthy adipose tissue, immune cells normally consist of CD4+ T-cell, regulatory T-cells (Treg), and type 2 macrophages (M2), which can regulate heat production, inflammation, and lipid metabolism. Nevertheless, in obese individuals, adipocyte hypertrophy and cytokine secretion result in a shift from M2 to M1 [50] and from Th2 to three different types: Th1, Th17, and CD8+ CTL. Obesity leads to increasing expression of the following: proinflammatory cells such as macrophages; integrins such as CD11b and CD11c; cytokines including TNF-*α*, IL-6, and nitric oxide synthase 2 (NOS2); and triggers such as Toll-like receptors (TLRs), metabolic endotoxemia, lipid spillover, and adipokines. These result in a shift from anti-inflammatory M2 type to proinflammatory M1 type [51]. The activation of NF-*κ*B pathways caused by these cytokines then ensues, thereby inducing the overexpression of proinflammatory cytokines such as TNF-*α* and IL-6. The activation of adipose tissue macrophages (ATMs) is an amplification of the inflammatory process [52]. In obese humans, long-term nutritional excess can lead to adipose tissue hypertrophy, adipose tissue vascularization, hypoxia, and adipose tissue necrosis. It can also lead to the infiltration of macrophages into adipose tissue and the surrounding necrotic tissue, which also produces a wide variety of proinflammatory cytokines [53]. In addition, the secretion of inflammatory cytokines and activation of the NF-*κ*B pathway trigger transcriptional expression of Nod-like receptor family pyrin domain containing 3 (NLRP3) and pro-IL-1*β* as well as the subsequent activation of NLRP3, causing macrophages to produce IL-1*β*. Overproduced saturated fatty acids in obese individuals can also stimulate NLRP3 inflammasome activation [54]. Inflammatory factors spread from adipose tissue into the blood circulation and then reach the lungs, which trigger airway inflammation and hyperresponsiveness. It has been reported that TNF-*α* can directly induce airway hyperresponsiveness (AHR) [55]. In addition, obesity can lead to abnormal fatty acid metabolism and increased fatty acid in blood by promoting increased expression of ACC1 and subsequently activation of ROR*γ*t to induce the differentiation of Th17 cells. IL-17 is produced by Th17 cells that bind with its receptors. IL-17 is able to improve the secretion of inflammatory cytokines such as IL-6, TNF-*α*, IL-8, CAM-1, and CM-CSF through the MAPK or NF-*κ*B pathway [56]. The association between obesity and neutrophil count in sputum is significant; in addition, a recent cluster analysis has shown that the presence of obesity-related asthma is characterized by increased airway neutrophils [57–59]. In addition to this, IL-17, IL-6, and TNF-*α* secreted by Th17 cells recruit and activate neutrophils in the lungs [60]. Neutrophils play an important role in ATM recruitment and inflammation by degradation of insulin receptor substrate 1 or activation of the TLR-4 pathway [61]. In addition to Th17 cells, Th1 cells in abnormal adipose tissue of obese individuals can secrete cytokines including IL-2, IL-3, IFN-*γ*, and TNF-*α*, which may activate M1 and thereby activate neutrophils through the chemokines (C-X-C motif) ligand 8 (CXCL8) pathway. Obesity-associated AHR was independent of adaptive immunity. A few eosinophils but many neutrophils were found in sputum, which suggests that obesity-associated asthma may be allergen independent [9]. Apart from the immune responses mentioned above, innate lymphoid cells also participate in the pathogenesis of obesity-related asthma. Although ILC2s have been reported to promote AHR and airway inflammation in a previous study [62], other studies showed that ILC2s played a crucial role in the repair of the airway epithelium, metabolic regulation, and translation from white adipose tissue into beige adipose tissue in the lungs [63]. However, a lack of ILC2 protection in obesity-related asthma may be partially due to the replacement of the ILC2 response with ILC3 response. In a study involving diet-induced obese mice, ILC3s and Th17 as well as the corresponding release of IL-17 were observed in the BALF, which may promote the development of AHR [64]. Bronchial epithelial cells stimulated by exogenous substances such as ozone [65] or cytokines can secrete IL-25, IL-33, and thymic stromal lymphopoietin (TSLP), thereby activating ILC2 to produce type 2 cytokines such as IL-4, IL-5, and IL-13. The pathway depends on the activation of the transcription factor GATA3 [66]. Macrophages, neutrophils, ILC2, Th1, and Th17 secrete a wide variety of cytokines and chemokines, which act on bronchial smooth muscle and may subsequently cause a series of asthmatic symptoms including airway narrowing, airway remodeling, mucus hypersecretion, AHR, airway smooth muscle constriction and hypertrophy, and a rapid decline in lung function.

### 3.2. Adipokine Secretion and Asthma

Obesity-related low-grade inflammation originates from the adipose tissue, which enables the secretion of a variety of interleukins and adipokines such as leptin, adiponectin, and resistin. In turn, these factors can affect obesity-related inflammation and airway inflammation [67]. Therefore, adipose tissue can be considered as a typical endocrine organ [68]. The amount of leptin produced by adipose tissue is higher in obese subjects compared to their nonobese counterparts [69]. However, a previous study found that leptin function in obese patients did not change. Leptin tolerance may be a causative factor in obesity-related asthma [70]. Moreover, overproduced leptin can stimulate the production of proinflammatory mediators, such as TNF-*α*, IL-6 from the adipose tissue, and IFN-*γ* from Th1 immune responsive CD4+ T-cells. TNF-*α* and IFN-*γ* are the mediators associated with AHR in asthma. On the other hand, leptin may inhibit the function and proliferation of regulatory T-cells, which may impair the balance of Th1 and Th2 and promote the polarization of Th1-mediated autoimmune diseases and Th2-mediated immune diseases such as asthma [71, 72]. Leptin not only affects the innate immunity but also has a significant impact on the allergic inflammatory response in obese individuals. Leptin promotes the proliferation and survival of proallergic Th2 cells and ILC2 by activating mTORC1, MAPK, and STAT3 pathways, which leads to the production of type 2 cytokines such as IL-4, IL-5, and IL-13, which together contribute to allergic responses [73]. In short, IL-4 promotes the production of IgE and subsequently activates mast cells; IL-5 activates and recruits eosinophils. IL-13 can directly act on goblet cells, which results in the secretion of airway mucus, airway remolding, and AHR [74]. A study found that the release of airway mucus induced by IL-13 can be regulated through the JAK2-STAT3-MUNC18b pathway [75]. There is no evidence that leptin is a direct cause of asthma. However, several studies have demonstrated that leptin is correlated with obesity and asthma in both adults and children [69, 76, 77]. Adiponectin is an anti-inflammatory adipokine produced by adipose tissue [78]. Adiponectin participates in inflammation by downregulating proinflammatory cytokines including TNF-*α*, IL-6, and NF-*κ*B and upregulating anti-inflammatory cytokines such as IL-1 receptor antagonists and IL-10. In addition, the decreased level of adiponectin reduces the inhibitory effect of bacterial lipopolysaccharide (LPS), thereby inducing macrophages to produce IFN-*α*, which induces the body's low-grade inflammatory reaction [79]. Adiponectin also induces a shift from M1 to M2 and from Th1 to Th2 and enhances the immunity and anti-inflammatory ability of body [80]. However, despite the enhanced inflammation in obese individuals, their levels of adiponectin are low [46]. This may result in the decreased anti-inflammatory activity of adiponectin. In addition, the overexpression of proinflammatory cytokines may inhibit the secretion of adiponectin, which results in further reduction in the anti-inflammatory effect of adiponectin, thereby providing the appropriate immunological environment for the onset and development of asthma. Furthermore, the effect of distinct isoforms of adiponectin varies in obesity-related asthma cases. A recent study found that the higher concentration of low-molecular-weight (LMW) adiponectin and the lower ratio of middle molecular weight (MMW) adiponectin/total adiponectin were evidently associated with asthma [81]. However, the level of high-molecular-weight (HMW) adiponectin in serum is not related to the onset and development of obesity-associated asthma [67]. Resistin, like leptin, is another proinflammatory adipokine produced by adipose tissue. Obesity is a chronic low-grade inflammatory condition accompanied with the increased production of various inflammatory factors including resistin. Moreover, inflammatory factors such as IL-1, IL-6, TNF-*α*, and LPS can promote resistin expression via NF-*κ*B-induced pathways. In turn, the proinflammatory cytokines including IL-6 and TNF-*α* can be promoted by resistin, thus decreasing obesity-associated inflammation [82]. These proinflammatory factors act on the lungs, which may lead to increased airway inflammation and asthma [83]. Through the comparison of serum leptin levels, Hassan et al. found that obese subjects with asthma showed higher resistin levels. With the increase in resistin levels, asthma severity is also accordingly increased [83]. Similarly, other studies have also demonstrated that the level of resistin and resistin/adiponectin ratio are proportionally increased in asthma and are even higher in obese subjects with asthma. In addition, the level of resistin and resistin/adiponectin ratio can also negatively predict lung function [67]. The above studies suggest that resistin can aggravate inflammation and promote the onset and deterioration of asthma. Thus, obesity may promote the occurrence of asthma and further aggravate asthma by promoting the increase of resistin. These studies probably uncovered a novel therapeutic target for obesity-related asthma.

### 3.3. Intestinal Microbiota Dysbiosis and Asthma

Intestinal microbiota mainly act as a biological barrier and are involved in immune regulation. Bacterial diversity increases mucosal immune defense [84]. However, obesity may result in intestinal microbiota dysbiosis and the reduction of bacterial diversity with an increase in Firmicutes and a reduction in Bacteroidetes [85], which are responsible for intestinal barrier and immune function damage. Thus, this results in weight gain, systemic inflammation, insulin resistance, and asthma [86]. Studies have reported that the concentration of LPS in plasma increased significantly in obese individuals, which may be due to the increase in intestinal permeability and excessive HFD [87]. Obesity-related low-grade inflammation and intestinal microbiota disorders can cause increased intestinal mucosal permeability [88]. LPS moves from the intestinal mucosa into the blood circulation, leading to endotoxemia. The related underlying mechanism is as follows: binding of LPS with TLR4 may activate the NF-*κ*B pathway, which produces a wide variety of cytokines, including TNF-*α* and IL-6 [89]. These cytokines act on the lungs, which may result in AHR and asthma exacerbation. Diet also affects the intestinal microbiota, thereby influencing asthma. A high-fiber/low-fat diet can increase the circulating levels of SCFAs [87] that play a critical role in inhibiting inflammation by regulating the differentiation and activation of Treg [90, 91], inhibiting LPS-induced NF-*κ*B activation, increasing TNF-*α* levels in neutrophils and macrophages, and inhibiting neutrophil production of proinflammatory reactive oxygen species (ROS) and TNF-*α* [92]. SCFAs stimulate intestinal epithelial proliferation and differentiation and could also contribute to the repair of the epithelial cell damage that is typical in asthma [92]. Moreover, SCFAs produced by the metabolism of dietary fiber from gut bacteria also promote the secretion of leptin to inhibit weight gain. However, a high-fat/low-fiber diet results in decreased levels of SCFAs and the imbalance of gut bacteria [93]. A study on an animal model reported that a low-fat/high-fiber diet promoted the production of beneficial bacteria such as Lactobacilli, Bifidobacteria, and Faecalibacterium and the production of SCFAs, especially butyrate. In contrast, the high-fat/low-fiber diet increased the Enterobacteriaceae, which are harmful to human health conditions [94]. High-fat/low-fiber diet-induced dysbacteriosis inhibits the function of regulatory T-cells through epigenetic modifications of the Forkhead box P3 (FoxP3) promoter and increases Th2-induced allergic airway inflammation. HFD-induced obesity may increase the risk for asthma via the changing gut bacteria. In addition, the obesity-induced dysbiosis of gut bacteria causes cholesterol metabolic disorders and the levels of bile acids are reduced in the gut, which impair the inhibition of NLRP3. NLRP3 activation induces IL-1*β* secretion primarily by M1 macrophage, which induces AHR that is considered as a major feature of asthma [95]. Many epidemiological studies have shown that with the increased number of caesarean section deliveries, there has also been an increase in obesity, type 1 diabetes, allergies, celiac disease, and some neurological disorders [96]. Caesarean-born children did not pass through the mother's birth canal, which causes intestinal microbial abnormalities, thus resulting in offspring obesity [97]. Studies found that compared with natural-born mice, caesarean-born mice gained more weight [98]. In addition, women who undergo caesarean section delivery usually use antibiotics preventively, which may be harmful to the baby's intestinal microbiota and may exacerbate obesity and other diseases. Another study found that ovalbumin-induced asthmatic mice had increased diversity of intestinal microbiota when exposed to microbes in early life. Moreover, IFN-*γ* levels and the ratio of IFN-*γ* and IL-4 were also increased significantly, which suggests that increased diversity of intestinal microbiota may induce Th1 response and inhibit airway inflammation in allergic asthma [99]. The results of these studies, taken together, indicate that a lack of microbial exposure in early life may cause obesity and asthma. Hence, we think that obesity caused by the lack of microbial exposure in early life may result in the risk and deterioration of asthma.

## 4. Potential Targeted Therapy of Obesity-Associated Asthma

There is a close association between obesity-associated asthma and several well-established risk factors for morbidity; thus, reversing the obesity-associated asthma is an urgent priority. We summarize herein potential targeted therapies for immune regulation, intestinal microbiota, and inflammation to control obesity-associated asthma (Figure 1). A better understanding of these different therapies will lead to future advances in the clinic.

### 4.1. Immune Regulator-Targeted Therapies

MicroRNAs (miRNAs), a class of noncoding RNAs, emerge as a novel treatment strategy for dyslipidemia and obesity via the modulation of sterols by SREBPs [100]. Among them, the most popular miRNA in the modulation of lipid metabolism is miR-33a, which is located in the intron of SREBF2. miR-33 regulates high-density lipoprotein (HDL) biogenesis and cholesterol efflux by downregulating the expression of ATP-binding cassette transporters [12]. miR-33 also targets SREBP1c, thereby affecting obesity [101]. A study using humanized knock-in mice observed the contribution of miR-33b to a relatively low HDL cholesterol level in human beings [101]. After induction by host SREBF genes, the miR-33 system facilitates lipid homeostasis by modulating the opposing regulator systems (plasma LDL versus HDL cholesterol and cholesterol versus fatty acid metabolism), indicating the therapeutic intervention of miR-33 in dyslipidemia [102]. Another example that has found clinical success in the regulation of lipid metabolism via SREBP is PCSK9 inhibitors, which indicate SREBP2 target PCSK9 in plasma and contribute to LDLR degradation [103, 104].

Intriguingly, extracts and natural compounds derived from some plants are also reported to possess antiobesity activity by targeting SREBP genes and other adipogenesis-related genes (such as PPAR*γ* and ACC1). Betulin, a natural triterpene isolated from the bark of birch trees, was reported to inhibit the maturation of SREBP by inducing the interaction of SREBP cleavage-activating protein (SCAP) and insulin-induced gene protein (INSIG), which lowered the biosynthesis of cholesterol and fatty acid. In vivo, betulin ameliorated diet-induced obesity by decreasing the lipid contents in serum and tissues and concomitantly enhancing insulin sensitivity. Thus, this is a potential compound for the treatment of hyperlipidemia and obesity [105]. Ginseng, which has been used in traditional Chinese medicine for centuries, is a novel depressor against SREBP and other transcriptional factors such as PPAR*γ* and ACC1. A South Korean research group found that compared with HFD mice, mice receiving HFD with Korean red ginseng extract (GE) (10 *μ*g/ml) for eight weeks had decreased body weight, adipose tissue mass, and adipocyte size, which indicated significant antiobesity effect [106]. Additionally, data in vitro confirmed that GE and two major ginsenosides, Rb1 and Rg1, inhibited adipogenesis by lowering PPAR*γ*, C/EBP*α*, and SCD1 expression at the gene level. Other studies have also reported significant antiobesity activity of Korean red ginseng by modulating transcription factors such as PPAR*γ* and aP2, which decrease the amount of lipid accumulation and inhibit adipogenesis [107–109]. These observations suggested that natural herbs are abundant medical sources for targeting SREBPs and other transcriptional factors in the treatment of obesity-related asthma.

In addition to inhibitors targeting the transcriptional factor SREBP, two independent research groups highlighted the potential clinical application of DPP-4 inhibitors in the attenuation of macrophage cell-mediated or immune cell-mediated inflammation [110, 111]. DDP-4 inhibitor or carotenoids such as *β*-cryptoxanthin and astaxanthin facilitate the immune regulation of lipid homeostasis in vitro at least partly by the decline in M1 macrophage numbers and the increase in M2 macrophage numbers, which indicate potential application in the treatment of obesity-related asthma in clinical and scientific studies [112, 113]. There was evidence demonstrating the role of Nrf2 in the treatment of obesity. A wide variety of synthetic Nrf2 inducers including triterpnoid 2-cyano-3, 12-dioxoolean-1, 9-dien-28-oic acid- (CDDO-) imidazolide, dithiolethione analog, and oltipraz demonstrated a significant ameliorative effect on HFD-induced obesity [114, 115]. However, it seemed that synthetic Nrf2 inducers were not clinically available due to the significantly increased risks of heart failure and the composite cardiovascular outcomes (nonfatal myocardial infarction, nonfatal stroke, hospitalization for heart failure, or death from cardiovascular causes) [116]. Sulforaphane, an isothiocyanate derived from cruciferous vegetables, is one of the most potent naturally occurring Nrf2 inducer. The compound was reported to ameliorate obesity by the enhancement of energy expenditure and the reduction of metabolic endotoxemia, which were caused by the decline in inflammation and insulin resistance. Sulforaphane may be a promising treatment for obesity-related asthma [29].

### 4.2. Intestinal Microbiota-Targeted Therapies

Gut microbiota have been associated with obesity-activating innate immunity through the LPS Toll-like receptor 4 axis [117]. Gut microbiota dysbiosis may induce obesity-related asthma [118]. We hypothesized that it may be beneficial to improve or treat obesity-related asthma via the modulation of gut microbiota. Moreover, the metabolic products of intestinal microbiota have a significant effect on body metabolism and immune defense mechanisms. Probiotics have a significant effect on improving intestinal microbiota modulation and systemic immunity. Previous studies indicated the weight of mice fed with HFD was the highest in three groups of mice with HFD, mice with HFD plus probiotics, and mice with HFD plus *Lactobacillus plantarum* (LP). The mice in high-fat diet plus LP exhibited significantly lower IL-6 and endotoxin (ET) content. Moreover, it will preferably regulate intestinal microbiota and systemic immune function to feed the combination of *Lactobacillus plantarum* (LP) and *Lactobacillus fermentum* (LF) [119]. In view of systemic obesity-related inflammation, the use of probiotics in the treatment of obesity probably has a potential effect on improving asthma. Clinical evidence demonstrated that the microbiome of obese people lacked a cluster of abundant Bacteroides species. Animal experiments showed that gavage with *B. thetaiotaomicron* can reduce serum glutamate concentration, which increased lipolysis and fatty acid oxidation process, thereby reducing fat accumulation and eventually achieving weight loss. Thus, it may be feasible to prevent obesity by regulating gut microbiota [120].

Obesity-related microbiota dysbiosis influences the production of bile acid, which is associated with lipid digestion and absorption [121]. Intestinal bile acids can be absorbed by bile acid binding resin (BAR) and likely improve obesity and metabolic disorders [122]. The binding of Berberine (BBR), extracted from the roots of *Rhizoma coptidis*, with gut and intestinal Farnesoid X receptor (FXR) lead to decreasing serum lipids in humans, hamsters, mice, and rats [123]. BBR inhibited bile salt hydrolase (BSH) activity in gut microbiota and activated FXR signaling pathway to alter bile acid metabolism, which may regulate lipid metabolism and then achieve weight loss. Therefore, BBR administration may be an effective strategy to improve obesity-related asthma [124].

Diet can also affect the intestinal microbiota balance and improve obesity and asthma. High-fiber diets can increase intestinal and circulating SCFA concentrations to suppress allergic inflammation and weight gain, which relies on the metabolism of intestinal microbiota [125]. Trompette et al. reported that high-fiber diet and SCFAs can shape immunological environment in the lungs by regulating the differentiation and activation of Treg. They can also affect the severity of allergic inflammation by inhibiting neutrophil production of proinflammatory reactive oxygen species (ROS) and TNF-*α* [90]. Thus, a diet with abundant dietary fermentable fiber may be beneficial for patients with obesity-related asthma. In addition, we summarized that increased activation of microbes producing SCFAs or the direct application of microbes producing SCFAs via fiber metabolism may also improve the symptoms of obesity-related asthma. Moreover, a prospective cohort study indicated that yogurt significantly prohibited weight gain, in particular among participants with higher fruit consumption, which may be due to a mechanism mediated by probiotics such as *Lactobacillus* and *Bifidobacterium* [126]. Additionally, gastrointestinal bacteria could metabolize foods such as beef, yogurt, and vegetable oils to produce conjugated linoleic acid (CLA) such as trans-10 and cis-12 CLA, which significantly prevented weight gain via mechanisms that increase adipocyte turnover and lead to the appearance of metabolically active beige adipocytes. Free linoleic acid and *α*-linolenic acid are converted to different CLA by *Bifidobacterium*, *Bifidobacterium pseudolongum* strain, and *Bifidobacterium breve* strains [127]. Therefore, CLA and CLA-associated compounds are a novel strategy to control weight that may be beneficial to improve obesity-related asthma.

### 4.3. Inflammation-Targeted Therapies

Obesity-related asthma is a chronic inflammatory disease accompanied with the disorder of proinflammatory and anti-inflammatory molecules driven by cytokines. Therefore, the regulation of cytokine secretion may be a new strategy to inhibit inflammation-induced obese-related asthma. MKP-1, a MAPK deactivator, can produce p38 MAPK phosphorylation in an irreversible manner, thereby inhibiting the occurrence of inflammation. Prabhala found that binding of dexamethasone or compounds interrupting the proteasome with MKP-1 can induce p38 MAPK phosphorylation and inhibit inflammatory cytokines in airway smooth muscle cells [128]. The interaction of p38 MAPK and MKP-1 may be a novel therapeutic target for obesity-associated asthma. Obesity-associated inflammation is mostly related to TLR4-NF-*κ*B pathway [129]. TLR4 knockout or antagonists may be beneficial for obesity-related asthma. TLR4 knockout relieves HFD-induced phosphorylation of IKK*β*, JNK, mTOR, and proinflammatory signaling molecules, which can alleviate obesity-associated inflammation. It might implicate the possible therapeutic potential of TLR4 in the management of asthma in HFD-induced obesity [130]. Furthermore, TLR antagonists have been used to treat metabolic diseases due to the beneficial effects of immune suppression in modern medicinal studies and applications. However, HFD-induced low-grade chronic inflammation may be an evolutional protective mechanism against pathogens. TLR antagonists or TLR knockout may inhibit the activity of host TLR, which probably increases the vulnerability to infection [131].

Chronic low-grade inflammation of the body induced by obesity also followed ILC2 activation. Cytokines derived from lung epithelial cells, such as IL-25, IL-33, and TSLP, can activate ILC2 to cause pulmonary injury. The activated ILC2s subsequently produce type 2 cytokines (IL-4, IL-5, and IL-13) and induce severe inflammation in the lungs [73]. Thus, inhibition of IL-25, IL-33, and TSLP possibly relieves type 2 inflammation. In a previous publication, a combination of an anti-TSLP antibody, AMG 157, and TSLP effectively obstructed the interplay between TSLP and its receptor, which may inhibit ILC2 activation [132]. Many clinical trials have revealed that antibodies against IL-5 or IL-5 receptor, IL-13, and IL-4R*α* modestly reduced asthma exacerbations and improved lung function [133]. Anti-interleukin-5 (anti-IL-5) is a neutralizing antibody targeting IL-5, which is essential for eosinophil maturation and survival. Two anti-IL-5 drugs have been approved by the Food and Drug Administration (FDA) in the US: mepolizumab [134], by subcutaneous injection monthly, and reslizumab [135], by intravenous infusion monthly. Ramirez-Carrozzi et al. concluded that commissural inhibition of IL-13 and IL-33 pathway or IL-5 and IL-13 pathway was extremely effective to reduce type 2 inflammation in patients with severe asthma [136]. Of note, the blockade of interleukin-13 using the two anti-interleukin-13 monoclonal antibodies, lebrikizumab and tralokinumab, potentially improved airway inflammation and smooth-muscle reactivity, which may reduce FENO but increase circulating eosinophil counts. The growth in peripheral blood eosinophil counts have been reported previously to reflect blocking of IL-13 activity [137]. In 2015, in a phase II clinical study of using lebrikizumab to patients with moderate-to-severe uncontrolled asthma despite ICS therapy and an additional controller, subcutaneous administration of lebrikizumab taken every four weeks reduced asthma exacerbation rate by 60% compared with placebo in periostin-high patients and by 5% in periostin-low patients. In this study, despite improving lung function, lebrikizumab treatments have not yet led to clinically meaningful placebo-corrected improvements in asthma symptoms or quality of life, potentially due to the limited power of the studies for these safety endpoints [138]. In 2017, the researchers had phase III clinical studies to provide further evidence of the safety and efficacy of lebrikizumab. Lebrikizumab, targeting IL-13 alone with biologics, has not shown a consistent reduction in asthma exacerbation. However, they confirmed that dupilumab, a medication simultaneously targeting at both IL-4 and IL-13 via blocking IL-4 receptor, has yielded more consistent results in reducing asthma exacerbations and improving lung function, especially in patients with increased blood eosinophils. Therefore, biologics targeting IL-4/IL-13 may be useful in patients with proof of T2-high asthma based on the presence of type 2 inflammation regardless of their baseline blood eosinophil levels [139]. Besides, late phase clinical trials of drugs targeting the IL-4/IL-13 pathways show promising results to achieve FDA-approved therapies. Doherty has said that TSLP can induce partial corticosteroid resistance, but under this condition, corticosteroid can still inhibit IL-33 to activate ILC2 [140]. Therefore, it is possible that corticosteroid can improve modestly obesity-related asthma. In addition, the function of ILC2 can be inhibited by Treg cells by the secreting cytokines of IL-10 and TGF-*β* in adipose tissue or via the direct contact of ILC2 with Treg, which can improve type 2 inflammation and deterioration of lung function. Therefore, taking effective measures to increase the number of Treg cells in asthmatic patients may reduce the inflammatory response induced by type 2 inflammatory cells [141]. In addition to ILC2, a team conducting experiments in mouse models of HFD concluded that NLRP3, IL-1*β*, and ILC3 cells facilitated obesity-related asthma by mediating inflammation [142]. In an experiment, after a short treatment of the IL-1*β* antagonist, anakinra, the symptom of AHR of obese mice induced by high-fat diets was improved [143]. Another team conducted a phase I clinical study, the result of which was that anakinra effectively reduced airway neutrophilic inflammation and caused no serious adverse events in a model of inhaled endotoxin LPS challenge. Thus, anakinra can be regarded as a potential therapeutic candidate for treatment of asthma with neutrophil advantage [144]. To date, it has not been investigated whether this type of treatment could be clinically applied in humans. In addition to inhibiting inflammatory cytokines, overall anti-inflammatory therapy may also be effective in obesity-related asthma. Similarly, obesity-related asthma frequently accompanies insulin resistance due to the lack of adiponectin. Calixto et al. conducted a study using obese mouse model fed with a HFD and found that metformin, a first-line treatment for diabetes, attenuated the exacerbation of the allergic eosinophilic inflammation [145]. In a retrospective cohort study, metformin users had a lower risk for asthma-related hospitalization and asthma exacerbation [146]. Hence, healthcare providers should consider metformin as a potential medication for patients with concurrent asthma and diabetes. It is well known that macrophages play an important role in the development and deterioration of obesity-related asthma and that peroxisome proliferator-activated receptors (PPARs) are expressed in monocytes/macrophages and adipose tissue. The activation of PPARs inhibits the shift from M2 to M1 and from Th2 to Th1 and also inhibits the secretion of proinflammatory cytokines such as IL-1*β*, IL-6, IL-10, IL-12, and TNF-*α* [147]. A study conducted by Yoon et al. demonstrated that the activation of PPAR-*γ* induced by apoptotic cell instillation over the course of bleomycin-induced lung injury can reverse the enhanced efferocytosis, the decreased expression of proinflammatory cytokines, and neutrophil recruitment, which likely inhibits inflammatory responses. Moreover, PPAR-*γ* activation may cause specific death of macrophages [148]. Therefore, PPAR-*γ* activation with PPAR-*γ* agonist or other PPAR-*γ*-stimulating compounds may reduce obesity-associated inflammation, thereby improving the severity of obesity-related asthma. To date, PPAR-*γ* agonists have not been studied clinically. Moreover, the regulation of adipokine level in obese individuals can also improve obesity-related asthma. Adiponectin secreted by adipose tissue is an anti-inflammatory adipokine and can promote the utilization of intracellular fatty acid and triglyceride-content reduction, whereas the level is decreased in obese individuals [149]. It has been demonstrated that adiponectin resistance observed in obese patients is due to the increased level of adiponectin in serum and the defective expression of adiponectin receptors in the lungs [150]. Despite some studies having shown no effect of recombinant adiponectin in animals, recombinant adiponectin may be a challenging therapeutic strategy for obesity-related asthma in the future.

## 5. Conclusion

As obesity becomes more prevalent worldwide, obesity-related asthma is frequently observed in the whole population. In this review, we described the disturbed lipid metabolism and immune modulators of lipid metabolism in obesity such as SREBPs, DPP-4, and Nrf2. In addition, we also discussed several immune factors potentially contributing to the pathogenesis of obesity-related asthma including intestinal microbiota, immune regulator, and inflammation. According to these possible immune causes in the onset and development of obesity-related asthma, we summarized several promising targeted therapies in the treatment of obesity-related asthma, such as miRNAs and TLR antagonists, which may provide effective medical intervention strategy in controlling obesity-related asthma.
